# Two Homologous EF-G Proteins from *Pseudomonas aeruginosa* Exhibit Distinct Functions

**DOI:** 10.1371/journal.pone.0080252

**Published:** 2013-11-08

**Authors:** Stephanie O. Palmer, Edna Y. Rangel, Yanmei Hu, Alexis T. Tran, James M. Bullard

**Affiliations:** The University of Texas-Pan American, Edinburg, Texas, United States of America; Louisiana State University, United States of America

## Abstract

Genes encoding two proteins corresponding to elongation factor G (EF-G) were cloned from *Pseudomonas aeruginosa*. The proteins encoded by these genes are both members of the EFG I subfamily. The gene encoding one of the forms of EF-G is located in the *str* operon and the resulting protein is referred to as EF-G1A while the gene encoding the other form of EF-G is located in another part of the genome and the resulting protein is referred to as EF-G1B. These proteins were expressed and purified to 98% homogeneity. Sequence analysis indicated the two proteins are 90/84% similar/identical. In other organisms containing multiple forms of EF-G a lower degree of similarity is seen. When assayed in a poly(U)-directed poly-phenylalanine translation system, EF-G1B was 75-fold more active than EF-G1A. EF-G1A pre-incubate with ribosomes in the presence of the ribosome recycling factor (RRF) decreased polymerization of poly-phenylalanine upon addition of EF-G1B in poly(U)-directed translation suggesting a role for EF-G1A in uncoupling of the ribosome into its constituent subunits. Both forms of *P. aeruginosa* EF-G were active in ribosome dependent GTPase activity. The kinetic parameters (*K*_M_) for the interaction of EF-G1A and EF-G1B with GTP were 85 and 70 μM, respectively. However, EF-G1B exhibited a 5-fold greater turnover number (observed *k*_cat_) for the hydrolysis of GTP than EF-G1A; 0.2 s^-1^ vs. 0.04 s^-1^. These values resulted in specificity constants (*k*_cat_^obs^/*K*_M_) for EF-G1A and EF-G1B of 0.5 x 10^3^ s^-1^ M^-1^ and 3.0 x 10^3^ s^-1^ M^-1^, respectively. The antibiotic fusidic acid (FA) completely inhibited poly(U)-dependent protein synthesis containing *P. aeruginosa* EF-G1B, but the same protein synthesis system containing EF-G1A was not affected. Likewise, the activity of EF-G1B in ribosome dependent GTPase assays was completely inhibited by FA, while the activity of EF-G1A was not affected.

## Introduction

EF-G is a member of the GTPase superfamily and functions in ribosome-dependent hydrolysis of GTP [1,2,3]. Early biochemical evidence indicated that EF-G is a multi-domain GTPase [4,5], an observation which has been confirmed by crystallographic data [6,7]. During protein synthesis, EF-G is involved in two distinct steps: elongation and ribosome recycling. During the elongation step, EF-G binds the ribosome and promotes the movement of tRNA and mRNA relative to the ribosome [8,9]. The relative shift of the mRNA is by a distance of one codon and the peptidyl- and deacylated-tRNAs are shifted from the pre-translocational to the post-translocational sites [9,10]. During the recycling step, EF-G acts in concert with the ribosome recycling factor (RRF) to effect the disassociation of the ribosome into its individual subunits [11,12].

EF-G was thought to exist exclusively in a single form as a bi-functional protein until recently when two genes (*hEFG1* and *hEFG2*) encoding two different forms of EF-G were discovered in mammalian mitochondria. Both of these were chromosomal genes encoding mitochondrial EF-G proteins: EF-G1_mt_ and EF-G2_mt_ [13,14]. EF-G1_mt_ has since been shown to be functional in the translocation step of protein synthesis [14]. EF-G2_mt_ lacks the ability to catalyze the translocation step in protein synthesis but in the presence of RRF can facilitate disassembly of the ribosome during the recycling event [15]. Analysis of 191 bacterial genomes in a study of ribosomal associated GTPases resulted in the identification of multiple forms of EF-G in up to 30% of the bacterial strains analyzed [16]. The second form of EF-G (EF-G2) has been isolated from three organisms and analyzed for activity. EF-G2 from *Thermus thermophilus* shows ribosome dependent GTPase activity, with little GTPase activity in the absence of ribosomes. It has a low level of activity in poly(U)-dependent protein synthesis but its role in ribosome recycling remains to be elucidated [17]. EF-G2 isolated from *Mycobacterium smegmatis* was assayed for ribosome-dependent GTPase activity and none was observed, indicating a lack of ability to function in either of the roles of EF-G under the conditions studied [18]. Only in *Borrelia burgdorferi* was the activity of both forms of EF-G studied in depth [19]. *B. burgdorferi* EF-G1 was found exclusively to act in translocation while EF-G2 was shown to function solely in ribosome recycling.

*Pseudomonas aeruginosa* is an opportunistic bacterial pathogen and the causative agent in a wide range of infections, including bacteremia, urinary tract infections, burn wound infections, and pulmonary infections in patients on respirators. A particularly serious medical problem caused by *P. aeruginosa* is chronic lung infection associated with cystic fibrosis [20]. In an attempt to better understand protein synthesis as it occurs in *P. aeruginosa*, we have cloned and over-expressed two forms of EF-G present in *P. aeruginosa*. The proteins encoded by these genes are both members of the EFG I subfamily [21]. The gene encoding one of the forms of EF-G is located in the *str* operon and the resulting protein is referred to as EF-G1A while the gene encoding the other form of EF-G is located in another part of the genome and the resulting protein is referred to as EF-G1B. Unlike multiple forms of EF-G from other organisms in which the amino acid sequence exhibits a low level of similarity, the amino acid sequence of the two forms of EF-G from *P. aeruginosa* are highly conserved. In this study, we compared the amino acid sequence of each of the EF-G molecules from *P. aeruginosa* and how they relate to homologous proteins from other organisms. We then showed ribosome-dependent GTPase activity, functionality in protein synthesis and the effect of fusidic acid (FA) on both forms of *P. aeruginosa* EF-G.

## Materials and Methods

### Materials

Oligonucleotides were from Integrated DNA Technologies (Coralville, IA). All other chemicals were obtained from either Sigma Aldrich (St. Louis, MO) or Fisher Scientific (Pittsburg, PA). Ribosomes from early log phase growths of *P. aeruginosa* strain PA01 were prepared in the laboratory of Walter Hill at the University of Montana (Missoula, MT) as previously described [22]. DNA sequencing was at the Howard Hughes Medical Institute (HHMI) laboratory at The University of Texas – Pan American. The plasmid pQE60-RRF(C-His) containing the gene encoding the *E. coli* ribosome recycling factor (RRF) was a kind gift from Dr. Nono Tomita-Takeuchi at the University of Tokyo (Kashiwa, Chiba, Japan).

### Gel Electrophoresis and Protein Analysis

Sodium dodecyl sulfate-polyacrylamide gel electrophoresis (SDS-PAGE) was performed using 4 to12% polyacrylamide precast gels (Biorad). Benchmark unstained protein molecular weight markers were from Invitrogen (Madison, WI). Protein concentrations were determined by the method of Bradford [23] using Coomassie Protein Assay Reagents (Thermo Scientific) and bovine serum albumin as the standard.

### Cloning and Purification of Two Forms of *P. aeruginosa* EF-G

Genes encoding both forms of EF-G were amplified by PCR (Bio-Rad MJ Mini Thermo Cycler) from *P. aeruginosa* PAO1 (ATCC) genomic DNA. EF-G1A was amplified using the forward primer (5’-ctgagctagcgctcgcaccactcccat-3’) and the reverse primer (5’-gactaagcttcatcagcggccctgcct-3’). EF-G1B was amplified using the forward primer (5’-ctgagctagcgcccgtactacacccatca-3’) and the reverse primer (5’-gactaagcttatcaaccttgttttttaaccagc-3’). The correct DNA sequence of PCR products was confirmed by DNA sequencing (Howard Hughes Medical Institute (HHMI) laboratory at The University of Texas – Pan American). The PCR products were inserted between the *Nhe*I/*Hind*III restriction sites in a pET-28b(+) plasmid (Novagen) and transformed into *E. coli* Rosetta™ 2(DE3) Singles™ Competent Cells (Novagen). This process placed the genes downstream of a sequence encoding six histidine residues.

Cultures were grown in F-medium (yeast extract,14 g/L, tryptone, 8 g/L, potassium phosphate-dibasic, 12 g/L, potassium phosphate-monobasic, 1.2 g/L and 1% glucose) containing 25 μg/ml of kanamycin and 75 μg/ml of chloramphenicol at 37 °C. Expression of the target proteins was induced at an optical density (A_600_) of 0.6 by the addition of isopropyl β-D-1-thiogalactopyranoside (IPTG) to 0.25 mM. Growth of the bacterial culture was continued for 3 h post induction and the bacteria were harvested by centrifugation (4000 x g, 60 min, 4 °C). The cells were lysed and Fraction I lysate was prepared as previously described [24]. Both forms of EF-G were precipitated between 45 and 60% saturation of ammonium sulfate and the precipitated protein was collected by centrifugation (23,000 x *g*, 60 min, 4 °C). Both forms of EF-G were further purified to more than 98% homogeneity using nickel-nitrilotriacetic acid (NTA) affinity chromatography (Perfect Pro, 5 Prime) followed by dialysis (two times) against a buffer containing: 20 mM Hepes-KOH (pH 7.0), 40 mM KCl, 1 mM MgCl_2_, 0.1 mM EDTA, 10 % glycerol. Purified proteins were fast frozen in liquid nitrogen and stored at -80 °C.

### Assays

Assays to determine the ribosome-dependent GTPase activity of EF-G were carried out in 50 μl reactions containing: 50 mM Tris-HCl, (pH 7.5), 10 mM MgCl_2_, 70 mM NH_4_Cl, 1 mM dithiothreitol (DTT) and 1.8 mM GTP. EF-G1A and EF-G1B concentrations were held constant at 1.0 and 0.3 μM in assays in which *P. aeruginosa* ribosomes were titrated and ribosomes were held constant at 0.4 μM in assays in which EF-G was titrated. Velocity assays contained indicated amounts of GTP and assays were stopped each min between 1 and 6 min. Assays were stopped by the addition of 150 μl of 50 mM ethylenediaminetetraacetic acid (EDTA). The amount of GTPase activity was determined by measurement of the amount of P_i_ liberated using a colorimetric GTPase assay kit (Novus Biologicals) per manufacturer’s directions. Fusidic acid (FA) effects were determined using the same assays but containing from 4 to 250 μM FA.

Protein synthesis assays were carried out in 50 μl reactions containing: 50 mM Tris-HCl, (pH 7.5), 10 mM MgCl_2_, 25 mM KCl, 4 mM phosphoenolpyruvate (PEP), 0.025 U/ml pyruvate kinase (PK), 1.5 mM ATP, 0.5 mM GTP, 40 μM [^3^H]phenylalanine (75 cpm/pmol), 0.3 mg/ml poly(U) RNA, 0.03 mM spermine, 1 mM DTT, 0.05 μM *P. aeruginosa* Elongation Factor-Ts (EF-Ts), 1.0 μM *P. aeruginosa* Elongation Factor-Tu (EF-Tu), 0.1 μM *P. aeruginosa* phenylalanyl-tRNA synthetase (PheRS), 0.2 μM *P. aeruginosa* ribosomes and the indicated amounts of EF-G1A or EF-G1B. Reactions were started by the addition of *E. coli* tRNA to a final concentration of 0.5 μM tRNA^Phe^ and continued for 1 h at 37 °C. Reactions were stopped by the addition of 2 ml 10% trichloroacetic acid (TCA) and filtered through glass fiber filters (Whatman) as previously described [25]. Retention of [^3^H]Phe represents the amount of poly-phenylalanine, poly(Phe), synthesized. The effects of FA on protein synthesis were determined using the same assays but contained from 4 to 250 μM FA. In these assays, the concentration of EF-G1A and EF-G1B were 1 μM and 0.2 μM, respectively. Assays to determine the effect of RRF on the activity of EF-G1B were the same as described above, with RRF titrated into the assay between 0.2 and 6.4 μM. Assays to determine the ability of EF-G1A/RRF to affect the activity of EF-G1B contained 0.2 μM EF-G1B, 1 μM EF-G1A and 2 μM RRF. In these reactions EF-G1A and/or RRF were pre-incubated in the reaction mix at 37 °C for 5 min, EF-G1B was then added and incubation was continued for 1 h.

## Results

### Sequence Analysis

**Figure 1:**
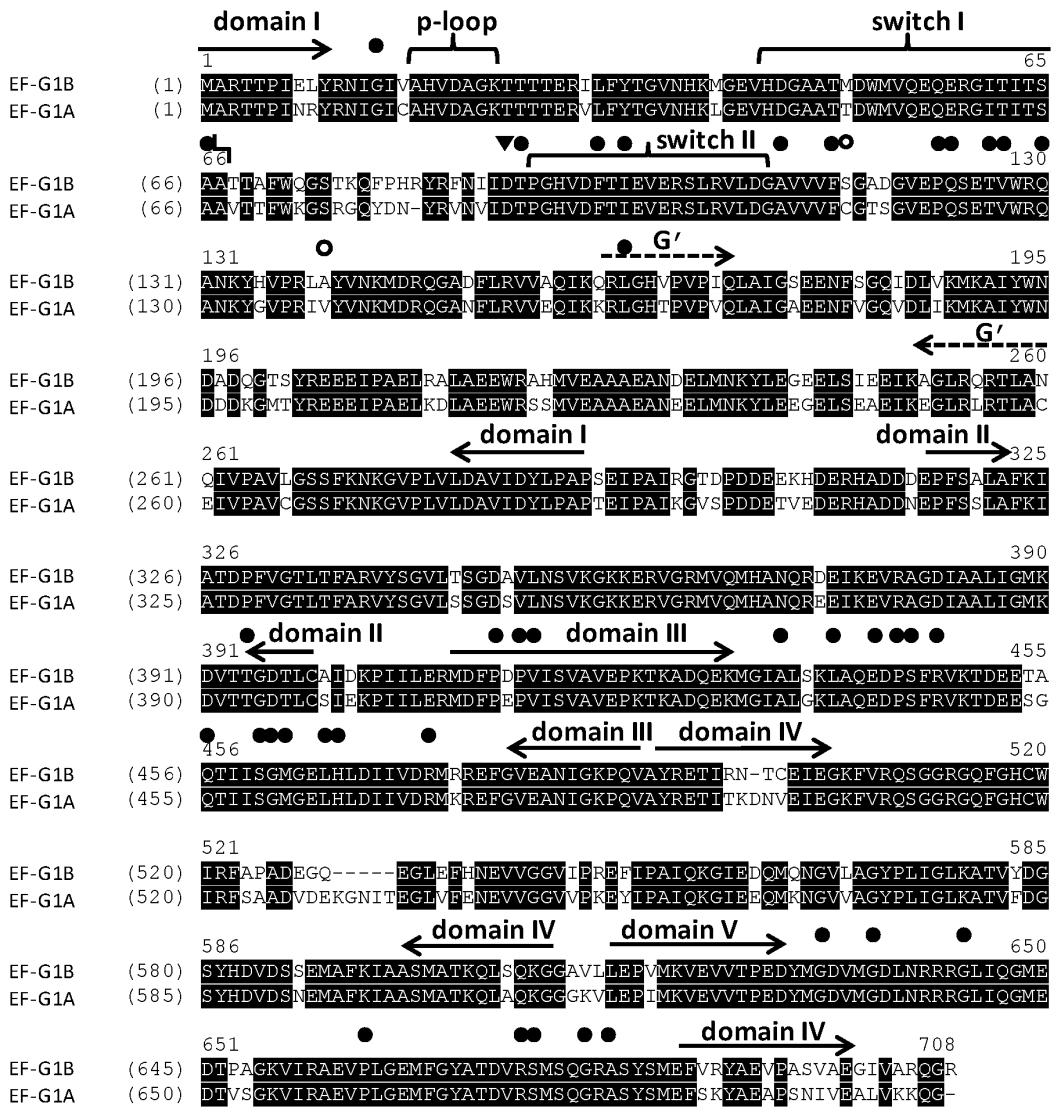
An alignment of EF-G1A and EF-G1B from *P. aeruginosa*. The protein sequences were downloaded from the National Center for Biotechnology Information (NCBI). The accession numbers for the two sequences are AAG05459 for EF-G1B and AAG07654 for EF-G1A. Sequence alignments were performed using Vector NTI Advance (TM) 11.0 (Invitrogen). Domains are designated with solid arrows and the G’ insert is designated with dotted arrows. Resistance mutations induced by fusidic acid at conserved amino acid residues are shown as closed circles (●) and mutations at invariant residues are shown as open circles (o). The amino acid representing the Walker B box is shown as (▼).

EF-G is a protein with a molecular mass of approximately 77 kDa. The crystal structure for EF-G from *T. thermophilus* has been solved bound to GDP and in the nucleotide free form; it appears to be an elongated protein composed of five structural domains [6,7]. More recently, the structure of *P. aeruginosa* EF-G1 was determined at 2.9 Å [26] and the structure was shown to be similar to that of *T. thermophilus* EF-G1. The structure of EF-G2 from *T. thermophilus* has also been determined and is similar to the structure to EF-G1 even though the sequence homology is only 30% [17]. Other organisms studied also indicate that there is only a modest level of overall amino acid sequence conservation between EF-G1 and EF-G2 proteins from the same organism (Table 1). In Table 1, the sequence identity of EF-G1 and EF-G2 from the same organism ranges from 29-56 % and the sequence similarity ranges from 44-68 %. Unlike the homologs shown in Table 1, the two EF-G-like proteins from *P. aeruginosa* (EF-G1A and EF-G1B) have a much higher level of amino acid sequence conservation; with the amino acid sequences being 84% identical and 90% similar (Figure 1). This is a much higher level of sequence conservation than observed when EF-G1 from different organisms are compared to each other and is similar to that observed when EF-G1 from different strains of the same bacteria are compared (data not shown). In the phylum Proteobacteria only two bacteria that were analyzed (*Bordetella bronchiseptica* and *Burkholderia rhizoxinica*) contained two EF-G molecules that contained a high level of amino acid sequence conservation. When *P. aeruginosa* EF-G1A and EF-G1B were compared to the four homologs from these two bacteria a high level of homology was observed in which the similarity only varied from 76 to 81% (Table 2). The EF-G molecules from *Bordetella bronchiseptica* and *Burkholderia rhizoxinica* were slightly more homologous to each other than either was with *P. aeruginosa* EF-G proteins, with the similarity only varying between 86 to 91%. This closer homology might be expected as both of these bacteria belong to the beta-subdivision of the Proteobacteria phylum while *P. aeruginosa* is a member of the gamma-subdivision.

**Table 1:** Conservation of amino acid sequence of EF-G isoforms from various organisms.

**Organism**	**Amino acid**	**Alignment**	**Accession Number**
	**(EF-G1/EF-G2)**	**(% similar/identical)**	
*Borrelia burgdorferi*	693/669 (24)^1^	51/34	YP_005806724/YP_005806873
*H. sapiens* (mitochondria)	751/777 (26)	44/32	NP_079272/AAH30612
*Mycobacterium semgmatis*	701/731 (30)	47/29	YP_006566130/YP_890748
*Thermus thermophilus*	691/658 (33)	46/30	YP_005300/YP_005103
*Bdellovibrio bacteriovorus*	701/702 (2)	57/41	YP_007023855/YP_007021899
*Bradyrhizobium sp.*	690/673 (17)	62/48	ZZ_10086950/CAL77572
*Mesorhizobium sp.*	696/683 (13)	47/31	YP_007305968/BAB49654
*Methylococcus capsulatus*	698/694 (4)	68/56	AAU_91593/YP_113063
*Shewanella sp.*	698/691 (7)	66/53	ABK46440/ABL98792
*Vibrio cholera*	698/695 (3)	65/53	NP_230015/NP_231972
*Pseudomonas aeruginosa* ^2^	702/706 (4)	90/84	AAG05459/AAG07654

^1^ The number in parenthesis represents the difference in the number of amino acids.

^2^ EF-G in *P. aeruginosa* are shown as EF-G1B/EF-G1A.

**Table 2:** Comparison of the conservation of amino acid residues from *P. aeruginosa* EF-G1A and EF-G1B with corresponding factors from other organisms.

**Organism**	**EF-G1B**	**EF-G1A**	**Accession Number**
	**(% similar/identical)**	**(% similar/identical)**	
*E. coli* ^1^ EF-G	80/67	78/67	0905186A
*B. burgdorferi* EF-G1	59/44	58/43	YP_005806724
*B. burgdorferi* EF-G2	55/36	55/36	YP_005806873
*H. sapiens EF*-G1_mt_	54/39	54/38	NP_079272
*H. sapiens EF*-G2_mt_	47/33	47/34	AAH30612
*M. smegmatis* EF-G1	71/58	70/57	YP_006566130
*M. smegmatis* EF-G2	45/29	44/28	YP_890748
*T. thermophilus* EF-G1	75/60	73/58	YP_005300
*T. thermophilus* EF-G2	51/33	51/32	YP_005103
*S. typhimurium* EF-G	80/69	79/69	AAL22309
*S. aureus* EF-G	72/59	71/57	A7WYX4
*B. bronchiseptica* (707) EF-G^2^	79/66	76/64	NP_890794
*B. bronchiseptica* (700) EF-G	81/67	80/66	CAE30528
*B. rhizoxinica* (703) EF-G	79/67	77/64	CBW74068
*B. rhizoxinica* (700) EF-G	79/66	78/66	CBW74549

^1^ The organisms analyzed are *Escherichia coli*, *Borrelia burgdorferi*, *Homo sapiens*, *Mycobacterium smegmatis*, *Thermus thermophilus*, *Salmonella typhimurium*, *Staphylococcus aureus*, *Bordetella bronchiseptica*, *Burkholderia rhizoxinica*.

^2^ The EF-G molecules from *B. bronchiseptica* and *B. rhizoxinica* could not be differentiated by sequence alignment and are differentiated here by the number of amino acids composing the proteins as shown in parenthesis.

When *P. aeruginosa* EF-G1A and EF-G1B were compared with EF-G1 and EF-G2 from other organisms, a wide variation in the amino acid sequence similarity was observed (Table 2). A higher degree of similarity was observed when *P. aeruginosa* EF-G1A and EF-G1B were compared with EF-G from organisms containing only one form of EF-G than with organisms containing multiple forms of EF-G. When compared with other bacteria having distinct EF-G1 and EF-G2 proteins, both *P. aeruginosa* EF-G1A and EF-G1B were more similar to EF-G1 than with EF-G2 (Table 2).

In a comparison of EF-G1A and EF-G1B, the functional regions (P-loop, switch I and switch II regions) of domain I are strictly conserved with only one residue variation from a methionine to a tyrosine (Figure 1). The five typical motifs (G1-G5) for GTP recognition [2] are also strictly conserved between EF-G1A and EF-G1B. EF-G proteins contain a region within the G-domain termed the G’ insert for which the functional significance is not well understood. It has been speculated that this region may be an internal guanine nucleotide exchange factor (GEF) [27], or possibly a region that specifically functions in ribosome binding [28]. The G’ insert is highly conserved between EF-G1A and EF-G1B, with only modest amino acid variations observed (Figure 1). In domain IV, at position 529 there is a five amino acid insert (KGNIT) observed in EF-G1A that is not present in EF-G1B. Results from alignments with all homologs analyzed indicate that three of these amino acids (N, I andT) are only present in *P. aeruginosa* EF-G1A (data not shown). These three residues are located in a loop region in domain IV and would therefore probably not affect function. The only region of lower sequence similarity is the region of domain IV at the C-terminus of the protein. This region contains the highest degree of divergence between EF-G1A and EF-G1B; however, the variations seen in these amino acids are moderate. In the structure of EF-G1 (EF-G1A) from *P. aeruginosa* this region forms an α-helix and is detached from the body of the protein [26].

Fusidic acid inhibits protein synthesis by trapping EF-G in the post-translocation step during elongation [29,30]. Mutations conferring resistance to FA have been mapped in EF-G from *T. thermophilus* [31,32], *S. typhimurium* [33] and *S. aureus* [34]. The positions that these mutations map to in EF-G from *P. aeruginosa* are shown in Figure 1. In the approximately forty sites in which mutations have been identified, all but two are strictly conserved between EF-G1A and EF-G1B, and these two sites vary from a Ser to Cys at position 115 (EF-G1B numbering) and from an Ala to Val at position 140 (Figure 1). None of the amino acid sequence differences observed would be expected to affect the function of the proteins significantly. Overall, from the amino acid sequence analysis and comparisons with EF-G from other organisms, the differentiation of the roles of EF-G1A and EF-G1B cannot be discerned.

### GTPase Activity and Inhibition by Fusidic Acid

**Figure 2:**
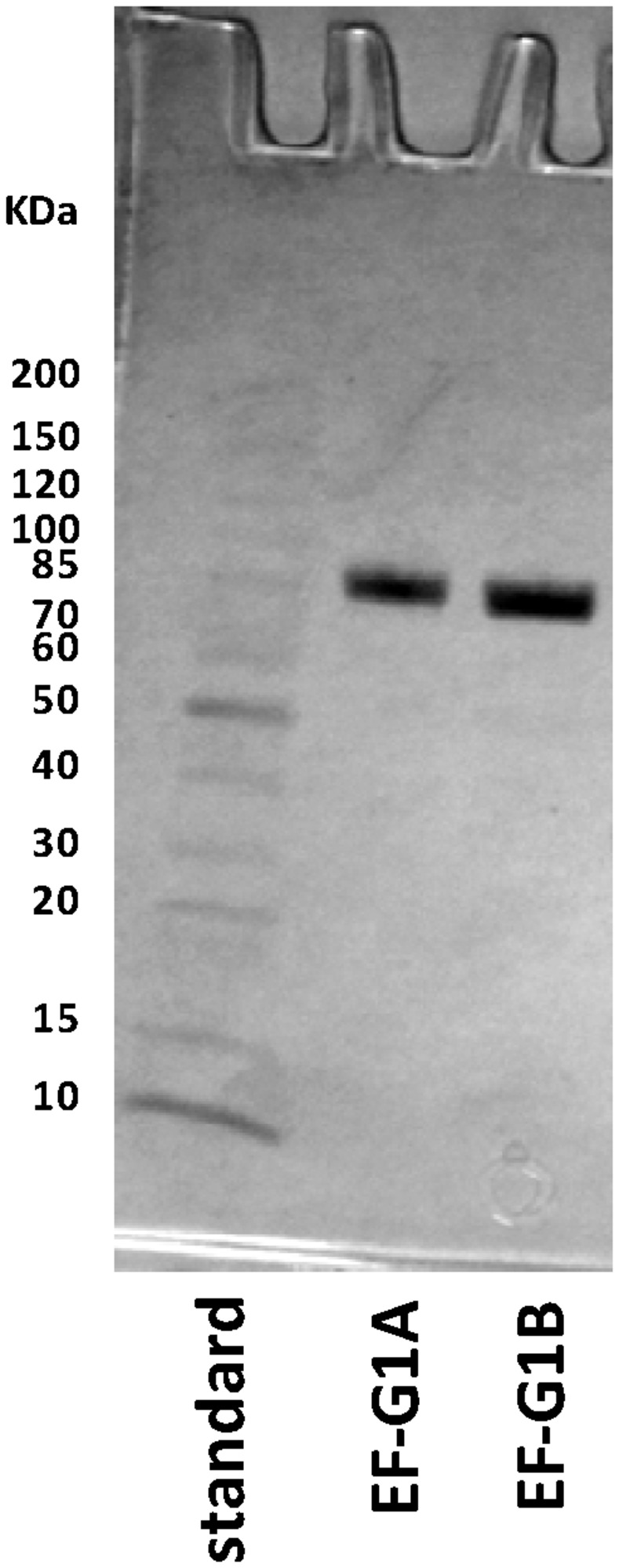
Sodium dodecyl sulfate-polyacrylamide gel electrophoresis (SDS-PAGE) analysis of purified *P. aeruginosa* EF-G1A and EF-G1B. Samples (1.0 μg) of the *P. aeruginosa* EF-G1A and EF-G1B preparations were analyzed on a 4-20% SDS-PAGE gel and the protein bands were visualized by staining with Coomassie blue.

**Figure 3:**
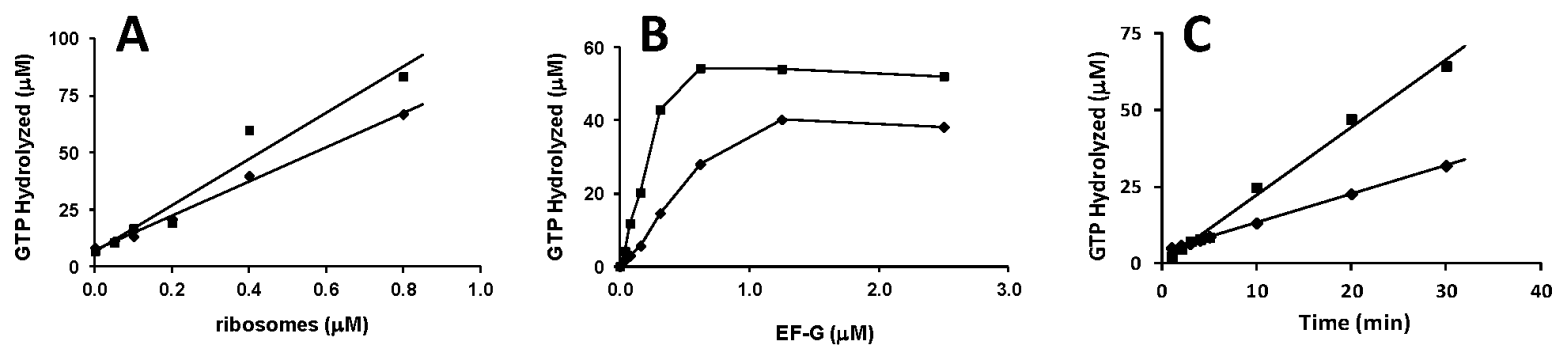
GTPase activity of *P. aeruginosa* EF-G1A and EF-G1B. Shown are representative GTPase assays of EF-G and ribosome dependence and a graph showing the linear increase in GTPase activity between 1 and 30 min. Assays are as described under “Methods and Materials”. A) Activity of EF-G1A and EF-G1B (1.0 and 0.3 μM, respectively) in the presence of varying concentrations of *P. aeruginosa* ribosomes. B) GTPase activity of EF-G at varied concentrations in the presence of 0.4 μM ribosomes. C) GTPase activity at increasing times. EF-G1A and EF-G1B are represented by filled diamonds (♦) and squares (■), respectively.

Two forms of EF-G from *P. aeruginosa* were cloned and expressed. The purification yielded both forms of EF-G in preparations that were greater than 98% homogeneous (Figure 2). GTPase activities of both forms of EF-G were shown to be dependent on the presence of ribosomes (Figure 3A). Only a low level of activity was observed in the absence of ribosomes and as the concentration of ribosomes increased, the level of GTPase activity increased. A ribosomal concentration of 0.4 μM was selected for downstream assays. When compared, EF-G1B exhibited approximately a 2-fold higher GTPase activity than was observed for EF-G1A (Figure 3B). At 0.5 μM (in the linear region of the plot), EF-G1A and EF-G1B were able to catalyze hydrolysis of 25 and 50 μM of GTP in 30 min reactions, respectively. At the inflection point on the titration curves EF-G1B was observed to have the same activity at 0.3 μM as EF-G1A had at 1.0 μM, therefore these concentrations were selected for downstream velocity assays at lower GTP concentrations. Timed assays showed that the GTPase activity of each form of EF-G was linear out to 30 min (Figure 3C).

**Figure 4:**
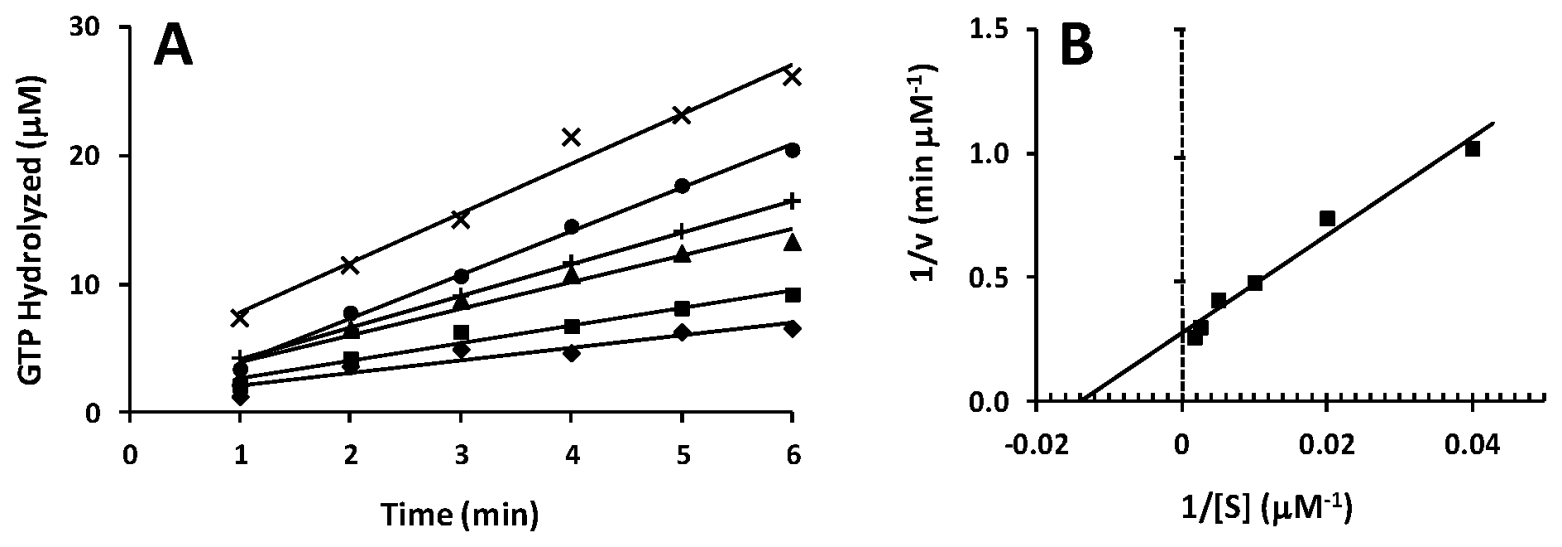
Determination of kinetic parameters for the GTPase activity of *P. aeruginosa* EF-G1B. A: Initial velocities for *P. aeruginosa* EF-G1B in GTPase activity reactions were determined at various concentrations of GTP. The concentration of EF-G1B was held constant at 0.3 μM. The velocities were measured between 1 and 6 min to minimize the chance of measurement of GTP hydrolysis occurring during mixing but before the beginning of the incubation period. The reactions were at 37 °C. The concentrations of GTP were: ♦, 25 μM; ■, 50 μM; ▲, 100 μM; +, 200 μM; ●, 400 μM, ×, 600 μM. B: The data from the initial velocity experiments were used to develop a Lineweaver-Burk plot to determine kinetic parameters for the GTPase activity of *P. aeruginosa* EF-G1B.

**Figure 5:**
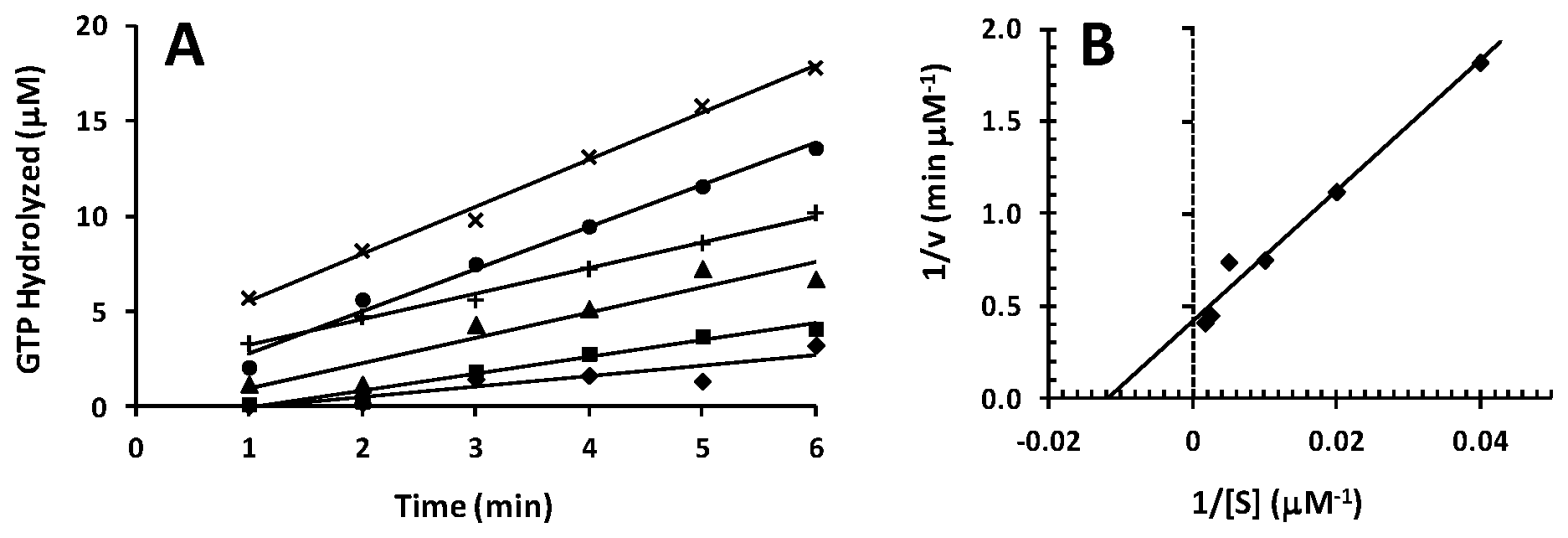
Determination of kinetic parameters for the GTPase activity of *P. aeruginosa* EF-G1A. A: Initial velocities for *P. aeruginosa* EF-G1A in GTPase activity reactions were determined at various concentrations of GTP. The concentration of EF-G1A was held constant at 1.0 μM. The velocities were measured between 1 and 6 min to minimize the chance of measurement of GTP hydrolysis occurring during mixing but before the beginning of the incubation period. The concentrations of GTP were: ♦, 25 μM; ■, 50 μM; ▲, 100 μM; +, 200 μM; ●, 400 μM, ×, 600 μM. B: The data from the initial velocity experiments were used to develop a Lineweaver-Burk plot to determine kinetic parameters for the GTPase activity of *P. aeruginosa* EF-G1A.

To determine the kinetic parameters governing the ribosome dependent hydrolysis of GTP by the two forms of *P. aeruginosa* EF-G, initial velocity assays were carried out at varying concentrations of GTP (from 25 to 600 μM). The kinetic parameters *K*_M_ and V_max_, for the GTPase activity of *P. aeruginosa* EF-G1B, were determined from Lineweaver-Burk analysis to be 71 μM and 3.7 μM/min, respectively (Figure 4). From these data the observed turnover number (*k*_cat_^obs^) was calculated to be 0.2 s^-1^ and the specificity constant (*k*_cat_/*K*_M_) was calculated to be 3.0 x 10^3^ s^-1^ M^-1^ (Table 3). The same procedure was used to obtain these parameters for EF-G1A (Figure 5). The *K*_M_ and V_max_ values were 85 μM and 2.4 μM/min, respectively, and the *k*_cat_^obs^ and *k*_cat_/*K*_M_ for the hydrolysis of GTP by EF-G1A were 0.04 s^-1^ and 0.5 x 10^3^ s^-1^ M^-1^, respectively. The *K*_M_ was observed to be very similar for both forms of EF-G; however, from these data the observed ability for the turnover of substrate (*k*_cat_^obs^) of EF-G1B is 5-fold greater than that of EF-G1A.

**Table 3:** The kinetic parameters governing the ribosome dependent GTPase activity of both forms of *P. aeruginosa* EF-G.

**EF-G**	***K*_M_**	***k*_cat_^obs^**	***k*_cat_^obs^/ *K*_M_**
	**(μM)**	**(s^-1^)**	**(s^-1^, M^-1^)**
EF-G1A	85	0.04	0.5 x 10^3^
EF-G1B	70	0.2	3.0 x 10^3^

**Figure 6:**
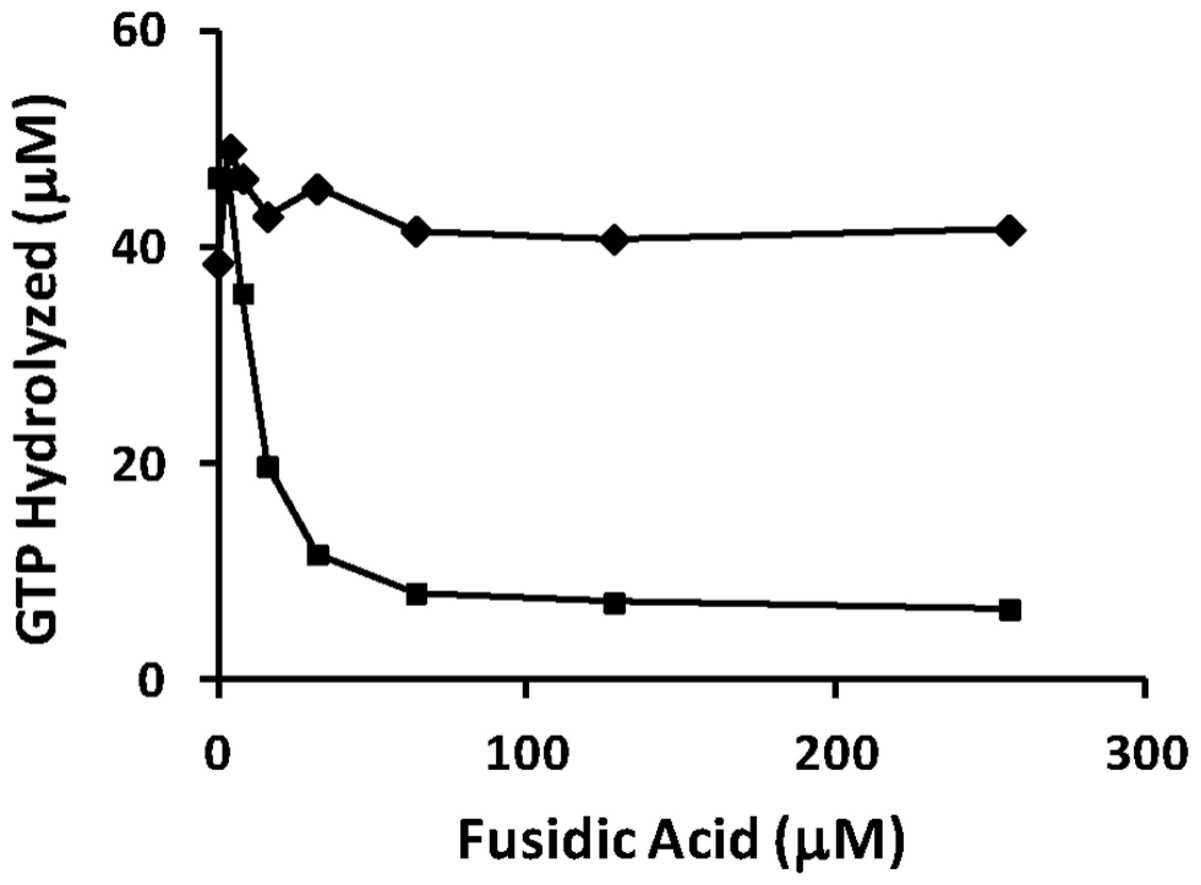
The effect of fusidic acid on the GTPase activity of *P. aeruginosa* EF-G1A and EF-G1B. Representative GTPase assays of the activity of EF-G1A and EF-G1B in increasing amounts of fusidic acid. The concentration of EF-G1A and EF-G1B were 1.0 and 0.3 μM, respectively, and the concentration of ribosomes was 0.4 μM. FA was added to the assay in concentrations from 4 μM to 250 μM. EF-G1A and EF-G1B are represented by filled diamonds (♦) and squares (■), respectively.

To determine the inhibitory effect of the antibiotic fusidic acid on the GTPase activity of both forms of EF-G, assays were performed containing FA at concentrations between 5 and 250 μM (Figure 6). The GTPase activity of EF-G1B was inhibited at the lowest concentration of FA tested and the activity was completely inhibited at FA concentrations above 30 μM. Alternatively, the GTPase activity of EF-G1A was not affected at any concentration of FA up to 250 μM.

### Function in Protein Synthesis and Inhibition by Fusidic Acid

**Figure 7:**
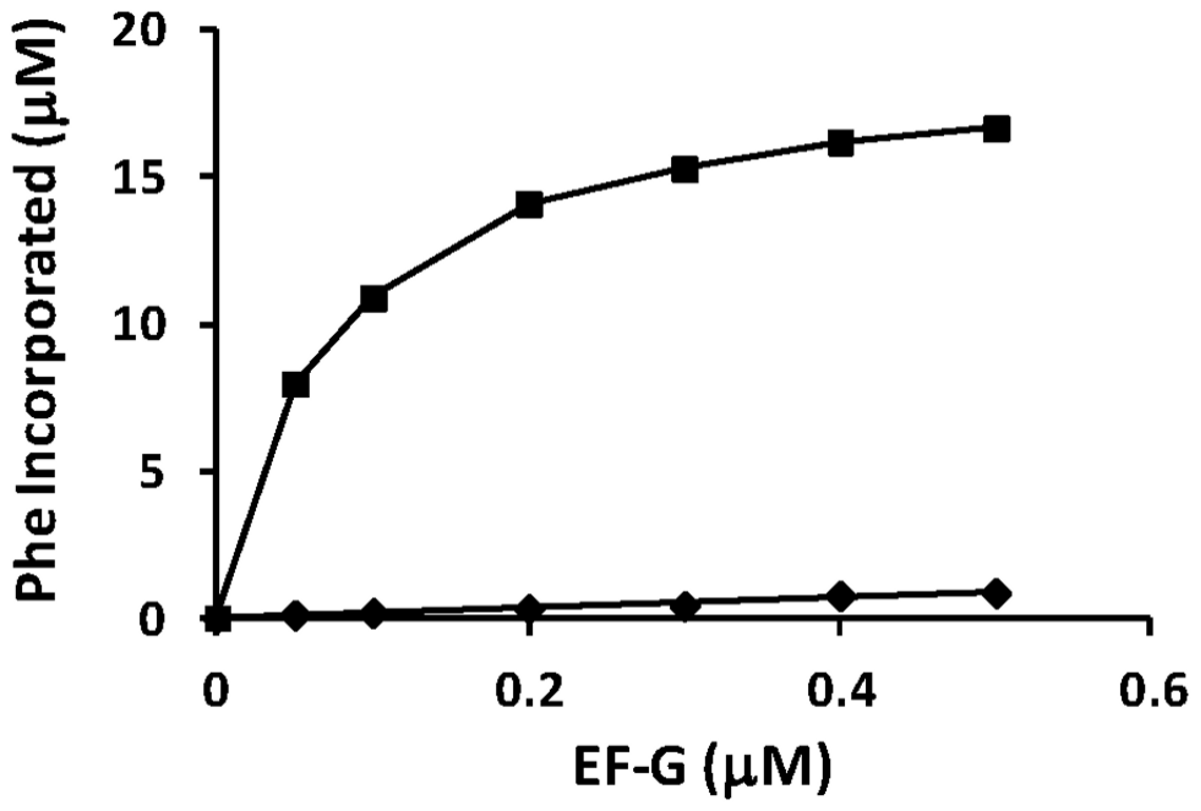
The ability of both forms of *P. aeruginosa* EF-G to function in protein synthesis. Representative protein synthesis assays containing increasing concentrations of EF-G1A and EF-G1B. The assays were as described under “Methods and Materials”. Concentrations of EF-G were as shown and concentrations of ribosomes were held constant at 0.2 μM. EF-G1A and EF-G1B are represented by filled diamonds (♦) and squares (■), respectively. “Phe Incorporated” represents the amount of phenylalanine incorporated into peptides during protein synthesis. Background activity (0.5 μM) was subtracted from the assay containing EF-G.

**Figure 8:**
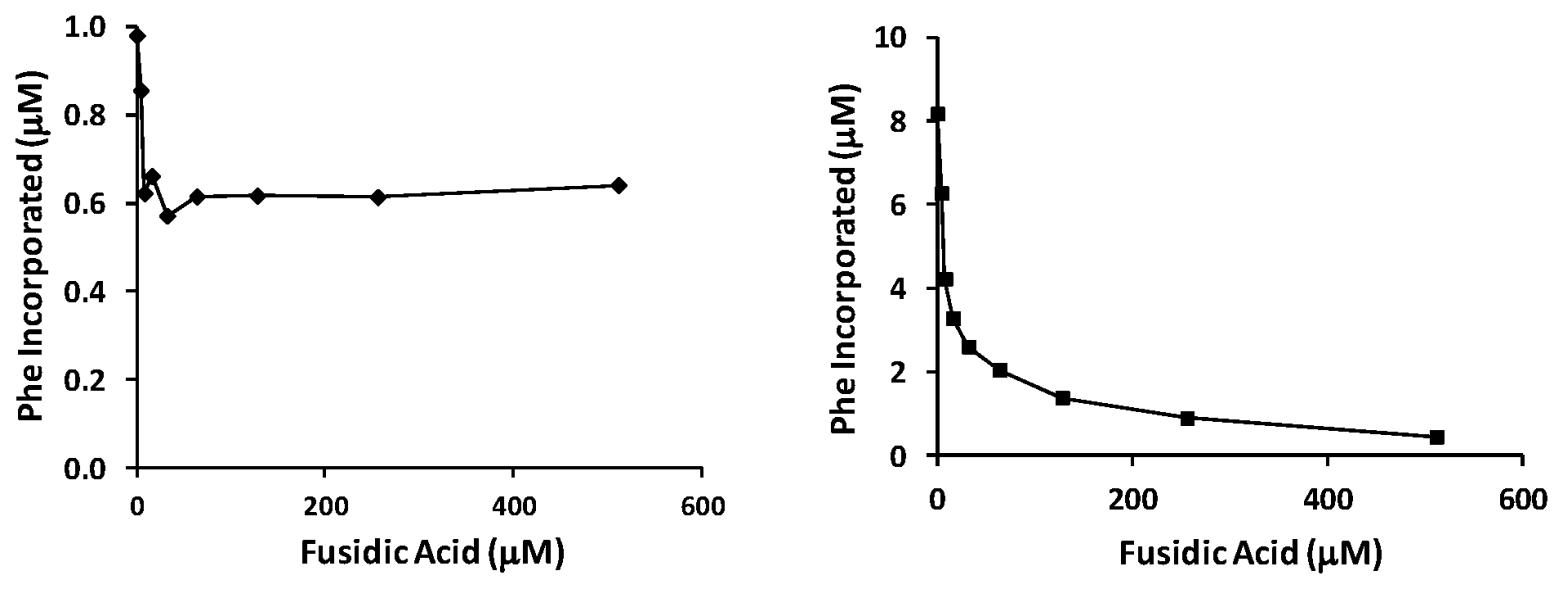
The effect of fusidic acid on the activity of *P. aeruginosa* EF-G in protein synthesis. Representative assays of the effect of increasing concentrations of fusidic acid on the ability of EF-G1A and EF-G1B to function in protein synthesis. A) EF-G1A and B) EF-G1B. The concentration of EF-G1A and EF-G1B were 1.0 and 0.2 μM, respectively, and the concentration of ribosomes was 0.2 μM. FA was added to the assay in concentrations from 4 μM to 500 μM. EF-G1A and EF-G1B are represented by filled diamonds (♦) and squares (■), respectively.

An aminoacylation/translation (A/T) system composed of *P. aeruginosa* protein synthesis components has been developed in our laboratory [35] (Figure 7). To determine the ability of both forms of EF-G to function in protein synthesis, each was tested for the ability to function in the synthesis of poly(Phe). Each form of EF-G was titrated into the assay between 0.05 and 0.5 μM (Figure 7). EF-G1B displayed robust activity in polypeptide synthesis. In contrast, EF-G1A was only observed to have a low level of activity. To ascertain the ability of FA to inhibit elongation, FA was added to protein synthesis assays at concentrations between 5 and 500 μM (Figure 8). As shown in Figure 8A, even though the activity of EF-G1A only yields 0.6 μM poly(Phe) in the elongation phase of protein synthesis, the addition of FA does not appear to affect the activity even at the highest concentration of FA. The initial drop in activity shown in Figure 8A is due to the inhibition of background activity, likely due to small amounts of EF-G1B co-purified with ribosomes. These results are similar to the lack of effect that FA had on the GTPase activity of EF-G1A. However, FA has a profound effect on the ability of EF-G1B to function in protein synthesis (Figure 8B). At the lowest concentration of FA (4 μM) the activity was reduced by 25% and at the highest concentration of FA the activity is completely inhibited. These results suggest that EF-G1B is involved in the elongation phase of protein synthesis and that EF-G1A is not.

### Ability of *P. aeruginosa* EF-G1A to Function in Ribosomal Recycling

**Figure 9:**
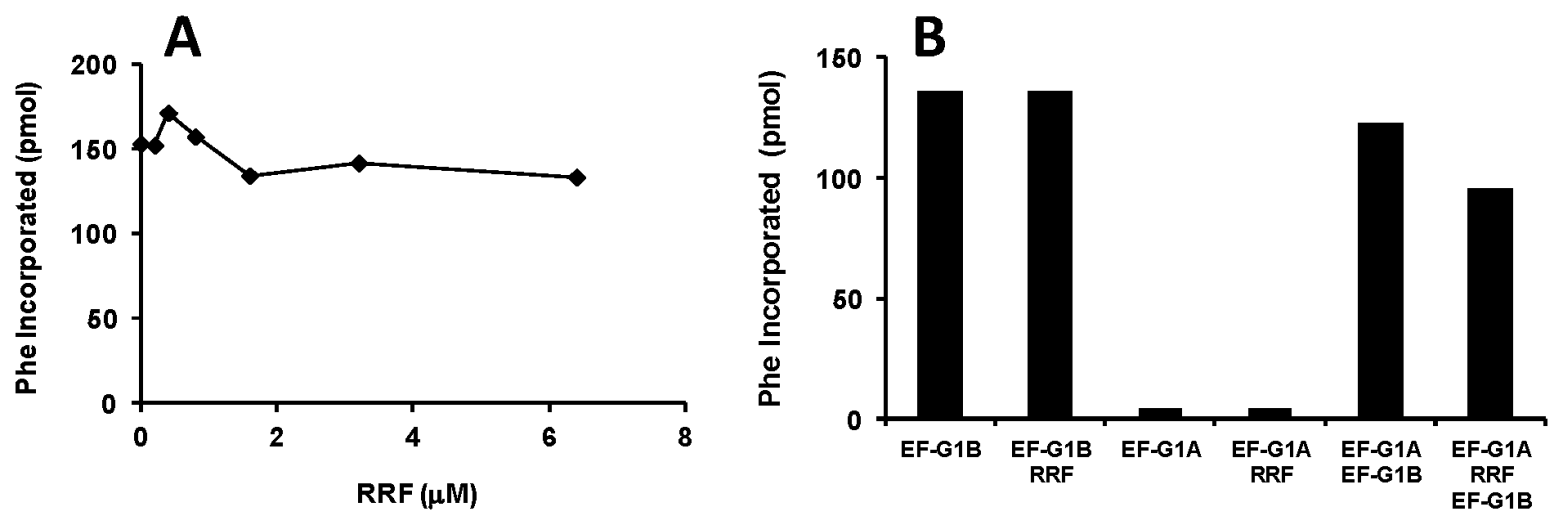
RRF and EF-G1A function to reduce the synthesis of poly(Phe). Representative assays depicting the effect of ribosome release factor (RRF) on the activities of EF-G1A, EF-G1B and a combination of EF-G1A and EF-G1B. A) Assays to determine the effect of RRF on the activity of EF-G1B were as described under “Methods and Materials”, with RRF titrated into the assay between 0.2 and 6.4 μM. The concentration of EF-G1B was 0.2 μM. B) Assays to determine the effect of RRF/EF-G1A on the activity of EF-G1B. The concentration of EF-G1B was the same as in “A” and the concentration of EF-G1A and RRF were 1 and 2 μM, respectively.

In mammalian mitochondria the two forms of EF-G were shown to have different functions. EF-G1_mt_ functions in the elongation stage of protein synthesis while EF-G2_mt_ in the presence of RRF functions in ribosomal recycling [14,15]. This was also shown to be the case in *B. burgdorferi* where EF-G1 was found to function in translocation while EF-G2 was shown to function exclusively in recycling [19]. To determine whether EF-G1A or EF-G1B functions along with RRF in catalyzing the separation of the two ribosomal subunits, each form of EF-G was tested in protein synthesis assays in the presence of increasing amounts of RRF (0.2 to 6.4 μM). Unlike protein synthesis using a natural messenger RNA, in poly(U) directed protein synthesis initiation can begin in the presence of tight-coupled (TC) 70S ribosomes [36]. If ribosomes are dissociated into the individual subunits the synthesis of poly(Phe) would be reduced. Likewise, the assay only detects poly(Phe) bound to ribosomes [37], therefore poly(Phe) synthesized and then released during the recycling event would not be detected and the overall activity would be reduced. When EF-G1B was assayed in this system (Figure 9A), no decrease in the synthesis of poly-Phe was observed at any concentration of RRF, indicating that EF-G1B was not able to act with RRF to recycle or separate the ribosomal subunits. In identical assays, the low level of activity of EF-G1A in protein synthesis was not observed to be affected by RRF at any concentration (data not shown). Figure 9B shows that RRF has no effect on the activity of either EF-G1A or EF-G1B in protein synthesis. When EF-G1A is added to the A/T protein synthesis system along with EF-G1B there is also no decrease in activity detected. However, when EF-G1A and RRF were pre-incubated with ribosomes prior to the addition of EF-G1B, protein synthesis was decreased by 25-30% (Figure 9B). These results indicate that EF-G1A and RRF act in concert to reduce protein synthesis. The recycling of the ribosomal subunits decreases the number of ribosomes available to function in protein synthesis and is the likely mechanism by which this is occurring.

## Discussion

We have expressed and characterized two forms of EF-G from *P. aeruginosa*. Both forms of *P. aeruginosa* EF-G appear to be more similar to homologs from bacteria containing only one form of EF-G than to those with multiple forms. When compared with homologs from bacteria containing more than one EF-G, both forms of *P. aeruginosa* EF-G appear more similar to EF-G1 than to EF-G2. Predictions from sequence analysis indicate that the two forms of the same protein would function equally well in protein synthesis. The amino acid residues comprising the primary sequences of the two forms of *P. aeruginosa* EF-G are highly conserved (90/84% similar/identical). Regions of the sequence that have been shown to be involved in hydrolysis of GTP are almost strictly conserved between the two forms. This is complemented by our data that show that the two EF-G molecules can both efficiently hydrolyze GTP in ribosome dependent reactions. If these findings are taken together, one could surmise that the two molecules are indeed different copies of EF-G performing the same role. This is supported by the fact that the many residues that have been shown to mutate in conferring resistance to FA are also almost strictly conserved between the two forms of EF-G from *P. aeruginosa* (Figure 1). However, at this point this line of thinking breaks down. FA traps EF-G in the post-translocation site on the ribosome after hydrolysis of GTP [29] and in this state any further GTPase activity stops. From our data we know that only one form (EF-G1B) of the two EF-G molecules is inhibited by FA. If this is true, the other form (EF-G1A) of the protein confers resistance to FA. However, if this is not the case then the form that is not affected by FA (EF-G1A) is not involved in translocation during the elongation phase of protein synthesis. Further experimentation indicated that only one form of the protein (EF-G1B) was functional in poly(Phe) synthesis (or the elongation phase) and this is the form that is susceptible to FA. This would weigh against the idea that the form of EF-G not susceptible to FA (EF-G1A) confers FA resistance. EF-G along with RRF functions in the disassembly of the post-termination ribosome [11]. Additional experimental evidence described here showed that the form of EF-G (EF-G1A) that is not susceptible to FA in the presence of RRF reduced the ability to synthesize poly(Phe). This provides preliminary biochemical evidence that EF-G1A may play a role in ribosome recycling; however biophysical experimental data would be required to definitively state that this is the case. This brings us to the conclusion that EF-G1B is the sole translocase in the elongation phase of protein biosynthesis in *P. aeruginosa*.

Evolutionary studies have identified four subfamilies of EFG: EFG I, spdEFG1, spdEFG2 and EFG II [21]. First, EFG I is encoded by the *fus* gene which is located in the *str* operon which also contains the genes encoding S12 and S7 ribosomal proteins along with a *tuf* gene which encodes EF-Tu (another protein involved in the elongation phase of protein synthesis). In many organisms this form of EF-G is the major translocase involved in protein biosynthesis. Next, the spdEFG subfamilies are restricted to three taxonomic divisions: *Spriochaetes, Planctomycetes and δ-proteobacteria* and to mitochondria [38]. The two subgroups of EF-G found in these three taxonomic divisions have been shown to have different functions in mitochondria and in *B. burgdorferi* [14,15]. Finally, EFG II proteins are highly divergent in primary sequence and thought to be a duplication of EFG I early in prokaryotic evolution which evolved along a different line [38]. *P. aeruginosa* EF-G1A and EF-G1B are both members of the EFG I subfamily. The *Pseudomonas* *sp.*, along with the *Burkholderia sp*.and *Bordetella* *sp.* are shown to have a second EF-G (located outside the *str* operon) that is termed a recent duplication from EFG I and the genes encoding these proteins are thought to have been acquired by lateral gene transfer [38]. The authors from this work [38] indicate that the high identity of these second EFG I proteins indicates retention of original function. We have shown here that the original function, at least in the case of *P. aeruginosa*, of the two highly conserved proteins has not been retained. *P. aeruginosa* EF-G1A is encoded by the *fus* gene located in the *str* operon and appears to function in ribosome recycling, while EF-G1B, which functions in translocation, is encoded in a secondary *fus* like gene (80% identical) and is located in the opposite side of the genome from the *str* operon. Perhaps as suggested for spdEFG1 and 2 [21], the duplication released EF-G from the constraints inherent to proteins with dual functions allowing each to become more specialized in distinct singular functions.

The structure of what is referred to as EF-G1 from *P. aeruginosa* has recently been solved by groups from Novartis [26]. This protein was called EF-G1 since it is encoded by the *fus*A gene that is located within the *str* operon. The natural compound argyrin B was shown to inhibit growth of *P. aeruginosa* and when the genome of one mutant was sequenced a single point mutation in the *fus*A gene (labeled as *fus*A1) was detected. The *fus*A1 gene was then sequenced from five additional *fus*A mutants and at least seven mutations (P414S, S417L, S459F, P486S, L663Q, T671A, and Y683C) were detected. There were no details from this work that indicated that the gene (*fus*A2) encoding the second EF-G (EFG 2) was sequenced from any other argyrin B resistant mutants after the initial genome that was screened. From the work described here, we know that the protein that was labeled EF-G1 (encoded by *fus*A1) is the form of EF-G (EF-G1A) that is not functional in elongation but appears to be involved in ribosome recycling. Comparison of the protein primary sequence indicates that the mutated residues identified in conferring resistance to argyrin B in EF-G1 (EF-G1A) are conserved in EF-G1B. Also, the residues in EF-G1 (EF-G1A) shown in the Novartis structure that directly interact with argyrin B are also conserved in EF-G1B. Another group [39] carried out a similar set of experiments and identified two additional mutation sites in the EF-G1 *fus*A1 gene, at positions Ile457 and Met685. The amino acids at these two positions are also conserved in EF-G1B. This group also sequenced the *fus*A2 gene (encoding EF-G1B) and found no mutations in argyrin B resistant mutants. The possibility exists that this compound, like FA, may inhibit the activity of one form of EF-G yet have no effect on the other form. If this is the case, argyrin B only inhibits the form of EF-G that may be involved in ribosome recycling. This study [39] also analyzed the level of mRNA encoding EF-G1 and EF-G2 from *P. aeruginosa* that was taken from clinical isolates. RNA transcripts encoding EF-G1 (EF-G1A) was observed to be more highly transcribed in clinical isolates than were transcripts encoding EF-G2 (EF-G1B). The sources of the clinical isolates were not given. This is contrary to what would be expected in actively growing cultures. In growing cultures, if EF-G1B functions exclusively in translocation and EF-G1A functions exclusively in ribosome recycling, since there are many more rounds of elongation than termination events one would expect to find more mRNA encoding EF-G1B than EF-G1A. However, if the samples were taken from respiratory passages in cystic fibrosis patients in which the bacteria form biofilms, a different set of mRNA might be expected. In biofilm formation the bacteria under goes a shift in behavior in which large suites of genes are differentially regulated [40]. In the anaerobic environment of cells making up the biofilm [41] many cells are in a stationary phase and growth is minimal therefore levels of mRNA encoding EF-G1B would be expected to be maintained at a low level.

In this work, we have shown that the two forms of EF-G from *P. aeruginosa*, which contain a high level of homology, perform distinctly different functions during protein biosynthesis. From an in-depth inspection of the primary amino acid sequences no viable reason for this was determined. It would be of interest in future studies to exchange the domains of the two forms of EF-G and determine which domain is responsible for the variation of activity as well as which region is responsible for the resistance to the antibiotic FA.
